# Supplementary material for: Synovial Sarcoma Chromatin Dynamics Reveal a Continuum in SS18:SSX Reprograming
Source: bioRxiv. 2024 May 17:2024.05.14.594262. Preprint. [Version 1] doi: 10.1101/2024.05.14.594262 (PMC11118320; doi:10.1101/2024.05.14.594262)

bioRxiv preprint doi: https://doi.org/10.1101/2024.05.14.594262; this version posted May 17, 2024. The copyright holder for this preprint (which was not certified by peer review) is the author/funder, who has granted bioRxiv a license to display the preprint in perpetuity. It is made available under aCC-BY-NC-ND 4.0 International license.

Supplementary Figures

Supplemental Figure 1. Synovial sarcoma samples do not have recurrent mutations. (a) Oncoplot displaying genes affected by missense mutations in more than one sample. (**b**) The

bioRxiv preprint doi: https://doi.org/10.1101/2024.05.14.594262; this version posted May 17, 2024. The copyright holder for this preprint (which was not certified by peer review) is the author/funder, who has granted bioRxiv a license to display the preprint in perpetuity. It is made available under aCC-BY-NC-ND 4.0 International license.

frequencies of the five overall most common single base substitution (SBS) signatures per case. (**c**) Whole genome copy number heatmap based on WGS data with samples ordered by the fraction of the genome altered (FGA). **(d)** Spearman correlation between the FGA calculated from WGS and WGBS data. **(e)** Whole genome copy number heatmap base on WGBS data with samples ordered by FGA. Abbreviations: MFS, metastatic free survival; Bi, biphasic; Mo, monophasic; PD, poorly differentiated; A, axial; DE, distal extremity; PE, proximal extremity.

Supplemental Figure 2. Active histone marks reveal two stable groups but do not have significant correlation with clinical outcome. Kaplan–Meier (KM) survival analysis of samples with or without (**a**) gain of chromosome 8, (**b**) gain of chromosome 12, or (**c**) loss of chromosome arm 3p. Unsupervised hierarchal clustering of the top 1 % most variable 500bp bins (distance = 1 - spearman correlation, ward.D2 clustering method) of genome wide (**d**) fractional methylation, (**e**) H3K4me3, (**f**) H3K4me1, (**g**) H3K9me3, (**h**) H3K27me3, (**i**) H3K36me3 and (**j**) H3K36me2 signal. KM analysis of the stable subgroups (bootstrap value > 70) observed in (**k**) H3K4me3, (**l**) H3K4me1, (**m**) H3K27ac. Abbreviations: DoD, dead of disease; Met, metastasis; Bi, biphasic; Mo, monophasic; PD, poorly differentiated; A, axial; DE, distal extremity; PE, proximal extremity; BP, bootstrap probability.

Supplemental Figure 3. Enhancer defined groups are not explained by SS18::SSX , cBAF, or GBAF levels. (a) Number of RNA-seq reads supporting the existence of the fusion per million mapped reads (fusion fragments per million, FFPM). (**b**) Miso analysis of the percentage spliced in (PSI) values for SS18 exon 8 isoforms between proximal and distal groups. (**c**) Western blot showing SS18::SSX and beta actin in HSSY2, U2OS (osteosarcoma cell line), 3

bioRxiv preprint doi: https://doi.org/10.1101/2024.05.14.594262; this version posted May 17, 2024. The copyright holder for this preprint (which was not certified by peer review) is the author/funder, who has granted bioRxiv a license to display the preprint in perpetuity. It is made available under aCC-BY-NC-ND 4.0 International license.

Proximal tumors (orange) and 3 Distal tumors (turquoise). (**d**) Quantified normalized expression of SS18::SSX compared to beta actin from the Western blot in HSSY2, U2OS (osteosarcoma cell line), 3 Proximal tumors (orange) and 3 Distal tumors (turquoise). (**e**) DAB nuclear optical density using antibodies for SS18::SSX, ARID1A, BRG1, PBRM1 and BRD9. (**f**) Western blot to demonstrate siRNA knockdown of SS18::SSX in SYO1 cell lines (5nM, 3 days). (**g**) Fraction of genome wide H2AK119Ub1 occupancy overlapping other histone marks in the C3H cell line expressing SS18::SSX and with SS18::SSX knockdown using CRE.

Supplemental Figure 4. Bivalency level correlates with cell cycle, core oncogenic, and immune cell expression signatures. (a) Spearman correlation between the number of bivalently marked promoters and the MYC expression. **(b)** Mean expression of SyS cell type specific signatures in the upper (Q4) and lower (Q1) bivalency quartiles. **(c)** Cibersort absolute values in bivalency high (upper quartile, Q4) and bivalency low (lower quartile, Q1) samples. Spearman correlation between the number of bivalently marked promoters and the mean expression of the SyS cell signature **(d)** "cell cycle" and **(e)** "core oncogenic program". **(f)** Spearman correlation between the number of bivalently marked promoters and promoters marked with H3K27me3 only. **(g)** Gene-gene correlation between the expression of the marker genes measured by RNA-seq and NanoString in the discovery cohort. **(h)** Receiver-operating characteristic (ROC) curve demonstrating that the NanoString signature was highly predictive of the bivalency groups in the discovery cohort (AUC = 0.957). (**i**) Proportion of samples falling within the FGA groups for bivalency quartiles. Boxplots of the number of **(j)** SNVs, **(k)** tumor size and **(l)** mean expression of SS18::SSX target genes for the upper and lower bivalency quartiles. * indicates *P*-value < 0.05 for a Welch two-sample t test.

bioRxiv preprint doi: https://doi.org/10.1101/2024.05.14.594262; this version posted May 17, 2024. The copyright holder for this preprint (which was not certified by peer review) is the author/funder, who has granted bioRxiv a license to display the preprint in perpetuity. It is made available under aCC-BY-NC-ND 4.0 International license.

Supplemental Figure 5. Synovial sarcoma has distinct promoter associated histone modification occupancy. Unsupervised hierarchal clustering (distance = 1 - spearman correlation, ward.D2 clustering method) of the proportion of promoter regions marked by (**a**) H3K4me1, (**b**) H3K27ac or (**c**) H3K27me3 in primary SyS (pink) and other normal and diseased tissue from the International Human Epigenome Consortium (IHEC).

Supplemental Figure 6. Synovial sarcoma has unique genome wide occupancy patterns of epigenetic marks. Genome wide occupancy of the histone marks **(a)** H3K4me1, **(b)** H3K27me3**,** **(c)** H3K27ac**,** **(d)** H3K9me3 and **(e)** H3K36me3 in SyS and control tissues. **(f)** H3K4me3 mean width of peaks overlapping SS18::SSX or SS18 wt binding sites. Mean fractional methylation of SyS and control tissues (**g**) genome wide, (**h**) in gene bodies and (**i**) intergenic regions. * indicates *P*-value < 0.05 using pairwise Wilcoxon signed-rank test.

Supplemental Figure 7. Synovial tumors have hypomethylated CGIs but are not sensitive to TET1 inhibition. **(a)** t-SNEA of beta values and fractional methylation values of SyS and other sarcoma subtypes. **(b)** Motif enrichment analysis in differentially hypomethylated regions. Mean methylation level of SyS and IHEC control tissues in **(c)** promoters harboring or (**d**) lacking CGIs. (**e**) Fraction methylation surrounding promoter bound CGIs in SyS and control tissues. (**f**) Expression of the *TET1* gene in SyS and IHEC control tissues. (**g**) Expression of the *TET1* gene in mouse and control samples. (**h**) Cell viability assay in SyS (HYSSII and SYO1) and Osteosarcoma (U2OS) cells upon 7-day treatment with 2-Hydroxyglutarate. (**i**) Cell competition assay performed in the synovial sarcoma lines HSSYII, SYO1, and Yamato

bioRxiv preprint doi: https://doi.org/10.1101/2024.05.14.594262; this version posted May 17, 2024. The copyright holder for this preprint (which was not certified by peer review) is the author/funder, who has granted bioRxiv a license to display the preprint in perpetuity. It is made available under aCC-BY-NC-ND 4.0 International license.

transduced with an empty sgRNA as control or with guides targeting TET1. * indicates *P*-value < 0.05 using pairwise Wilcoxon signed-rank test.

Supplemental Figure 8. WDR5 is a selective vulnerability in synovial sarcoma. (**a**) Interrogation of RNAi viability scores for a panel of MLL and COMPASS complex members in SyS versus all other cell lines presented in the DepMap database identifies WDR5 as a selective vulnerability. Red dots indicate SyS cell lines and the black line within the violin plot represent the mean score for all cell lines. (**b**) Expression of the MLL and COMPASS complex members in SyS. (**c**) CRISPR CERES scores for a panel of MLL and COMPASS complex members in SyS versus all other cell lines presented in the DepMap database.

Supplemental Figure 1

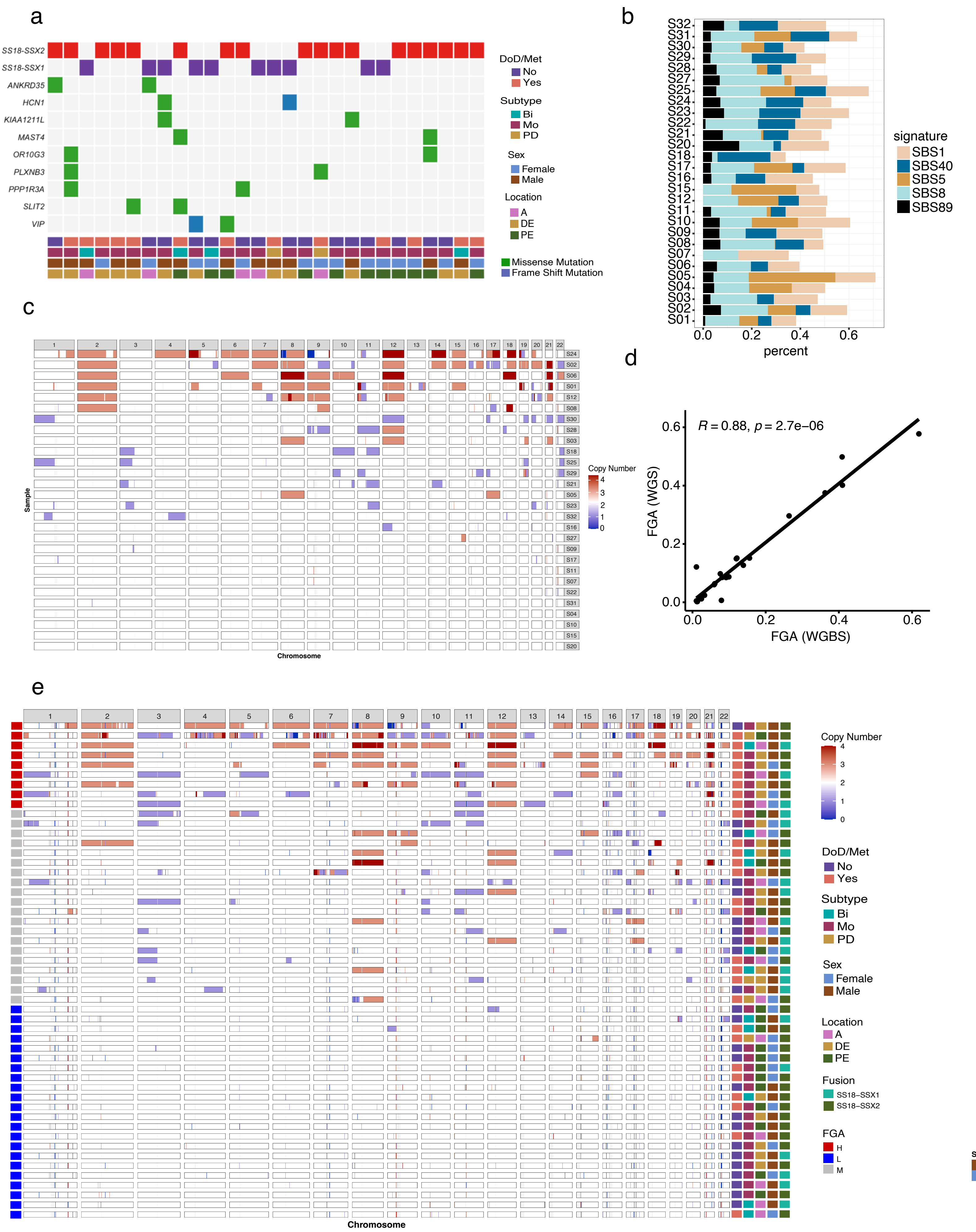

Supplemental Figure 2

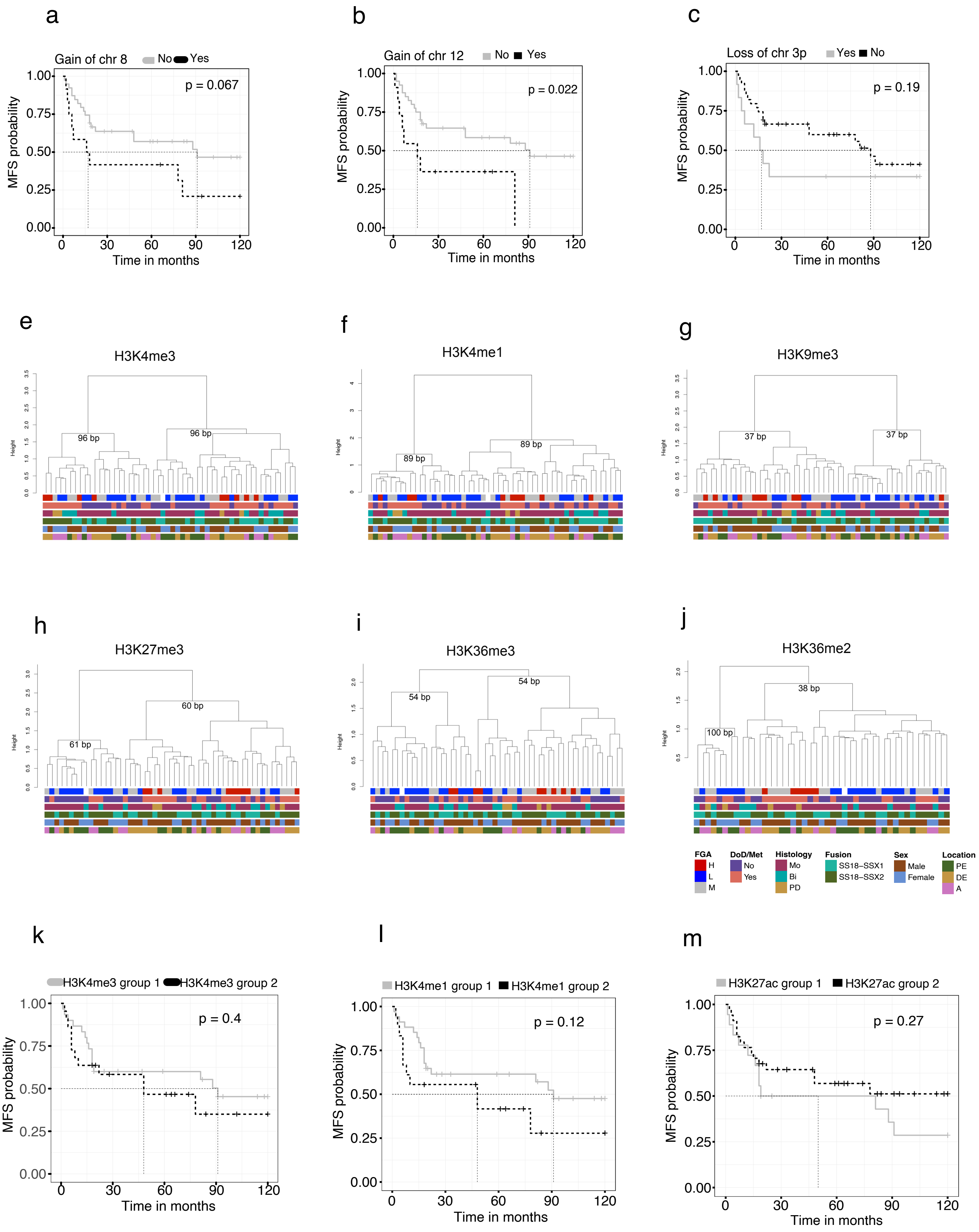

Supplemental Figure 3

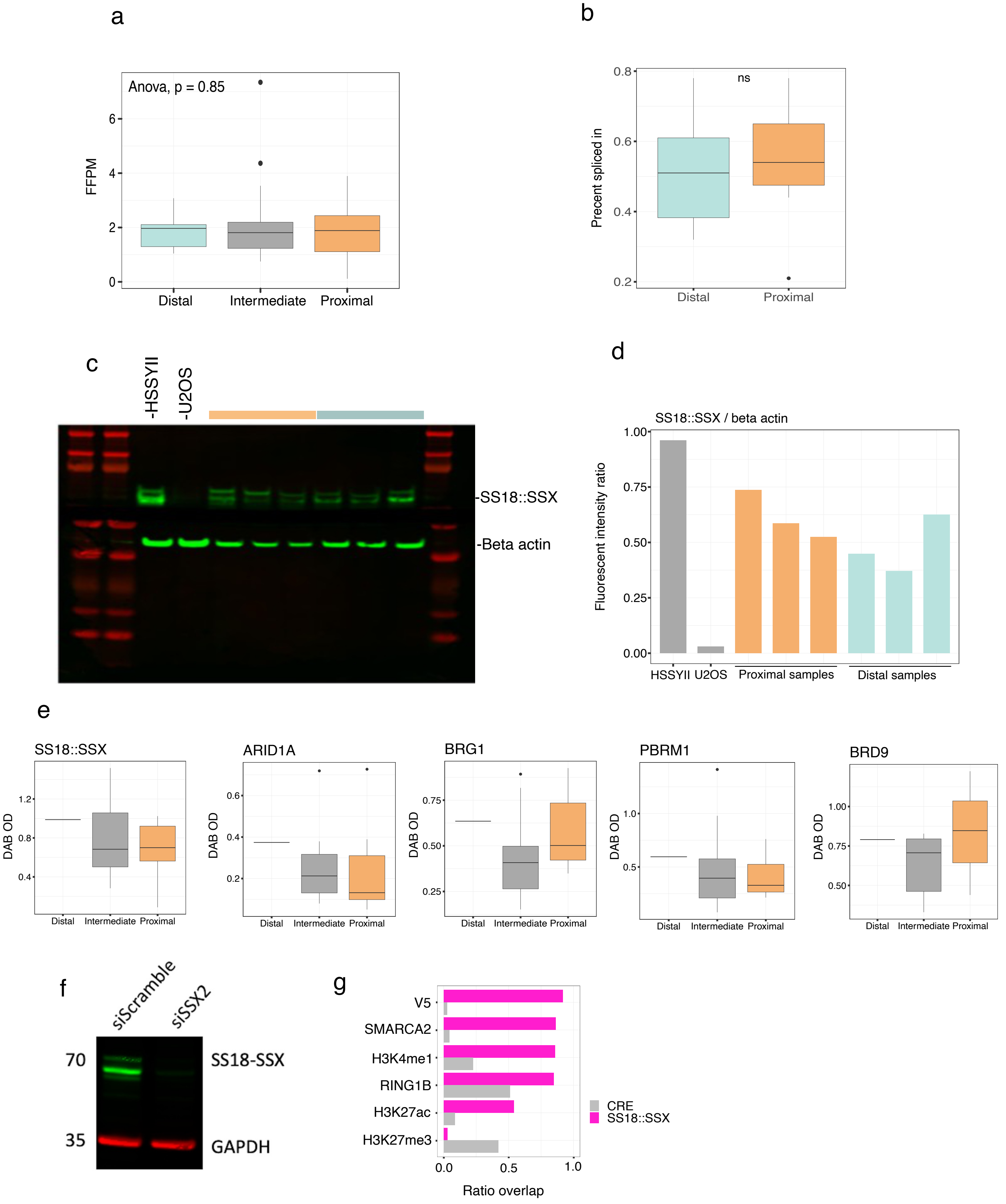

Supplemental Figure 4

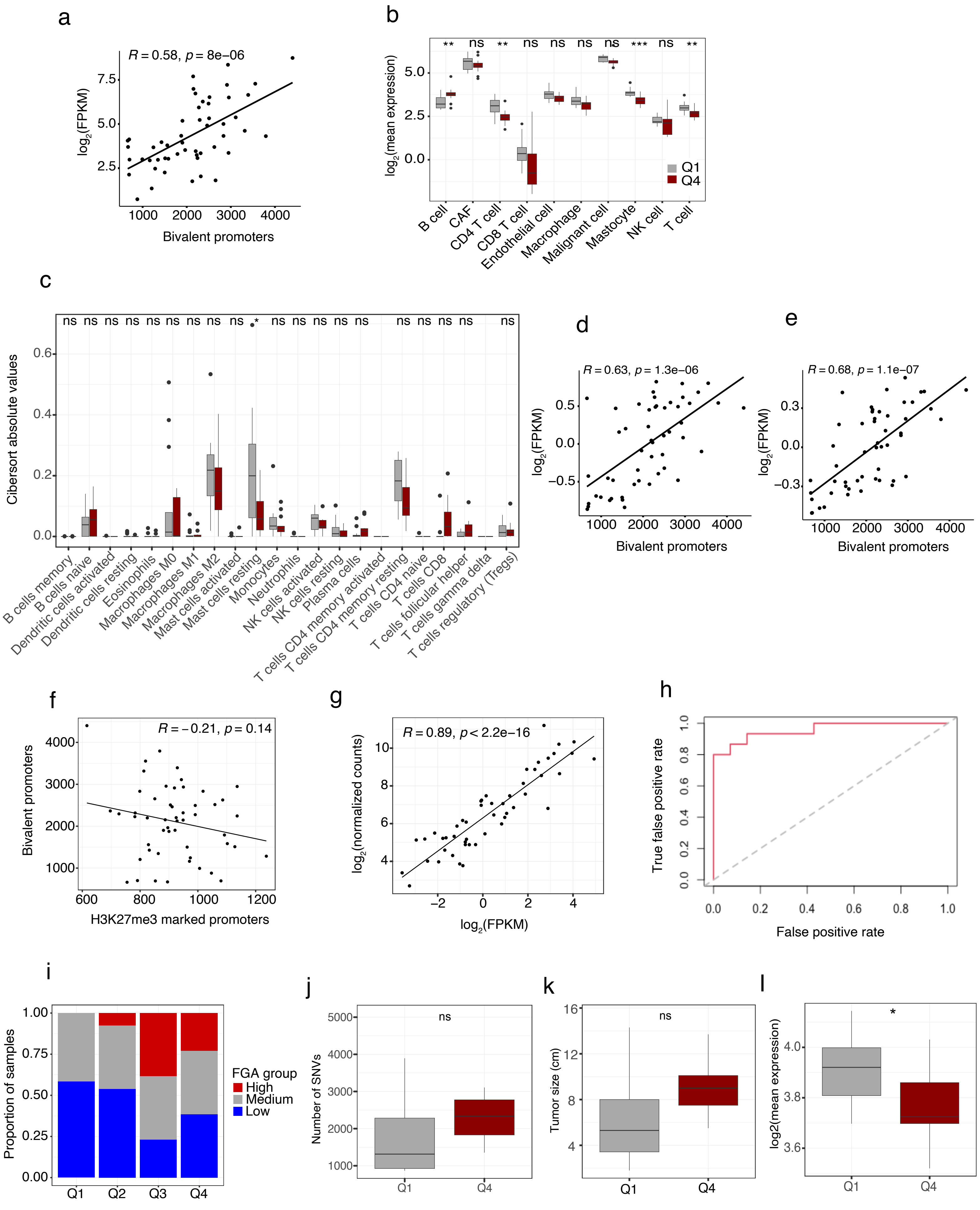

Supplemental Figure 5

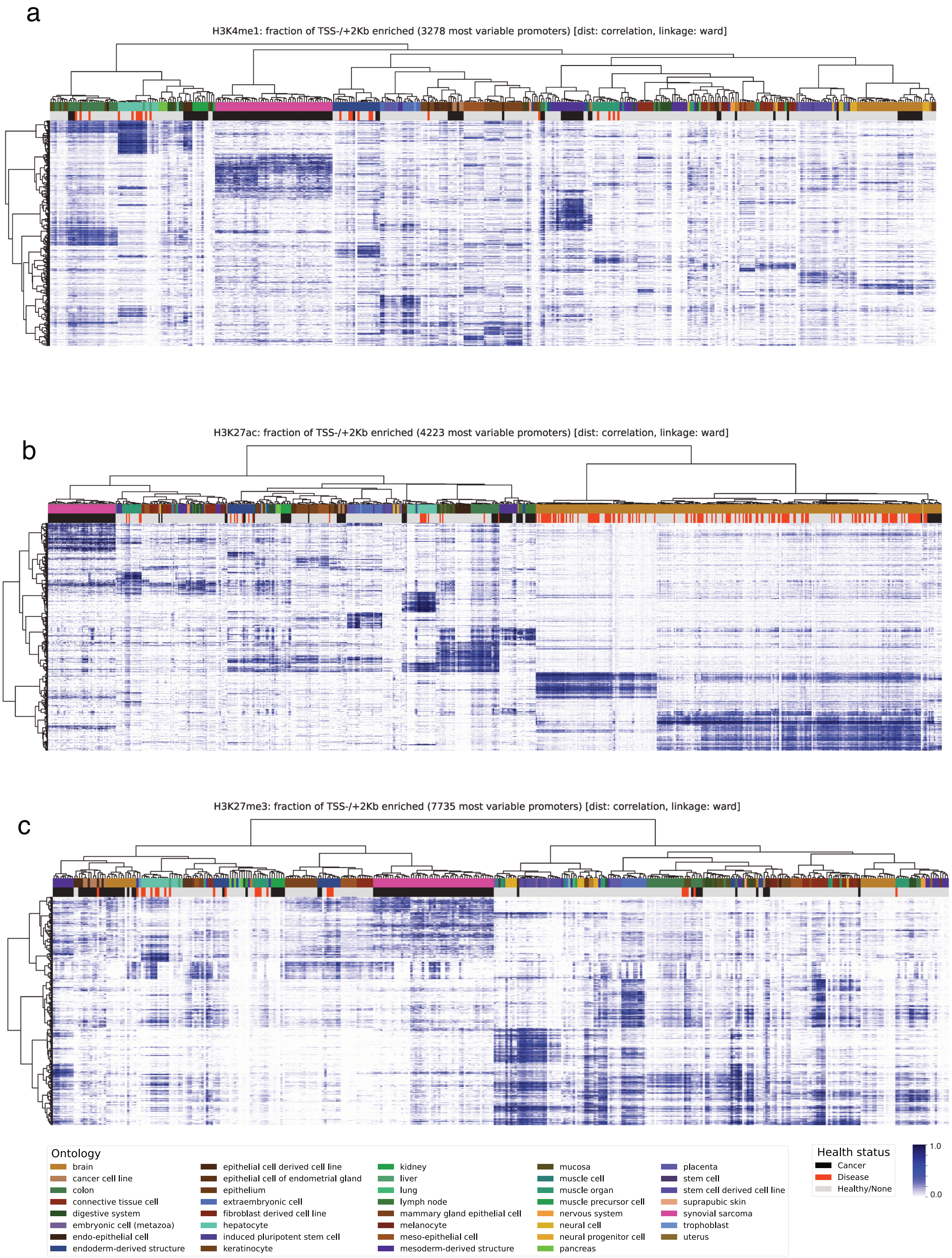

Supplemental Figure 6

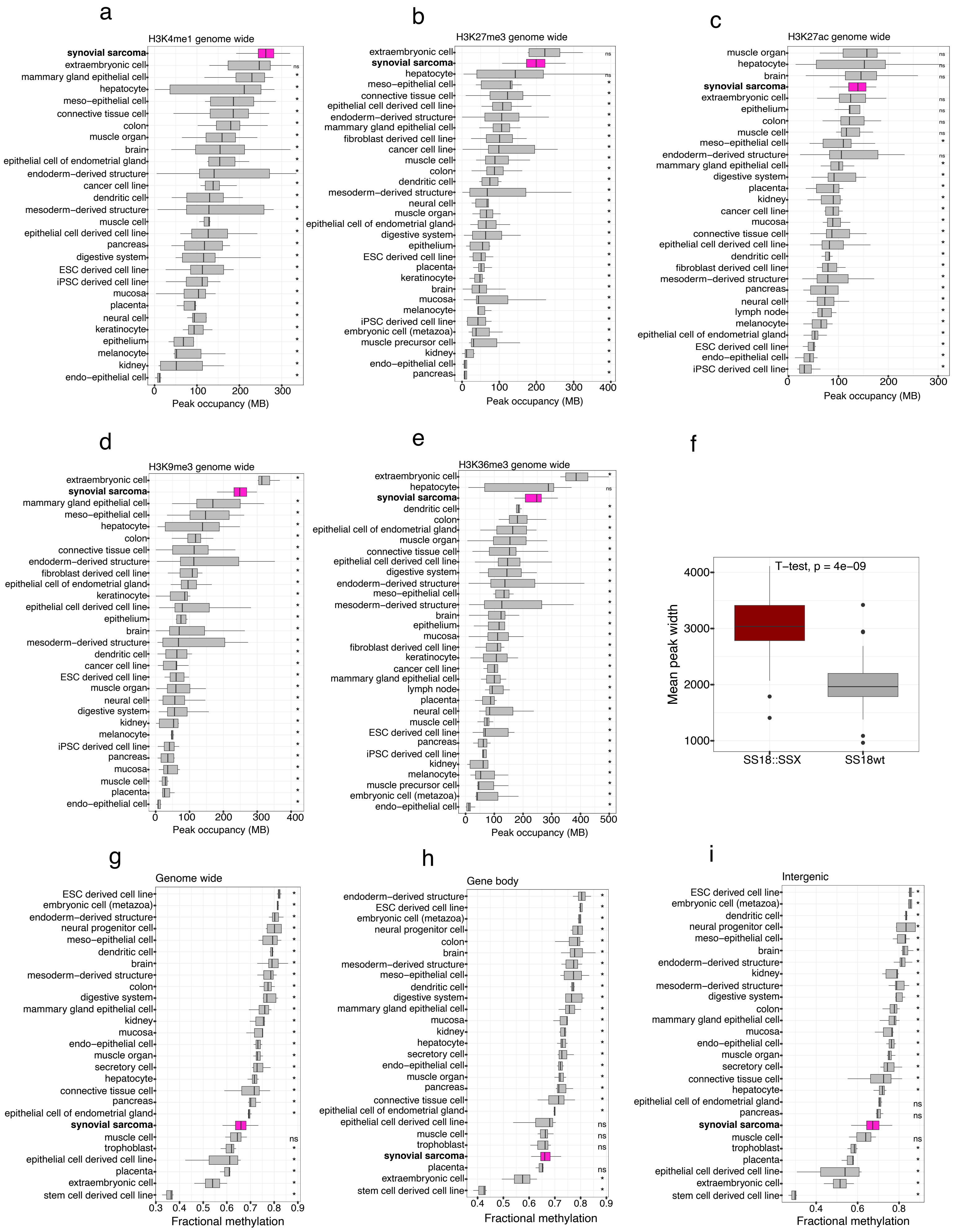

Supplemental Figure 7

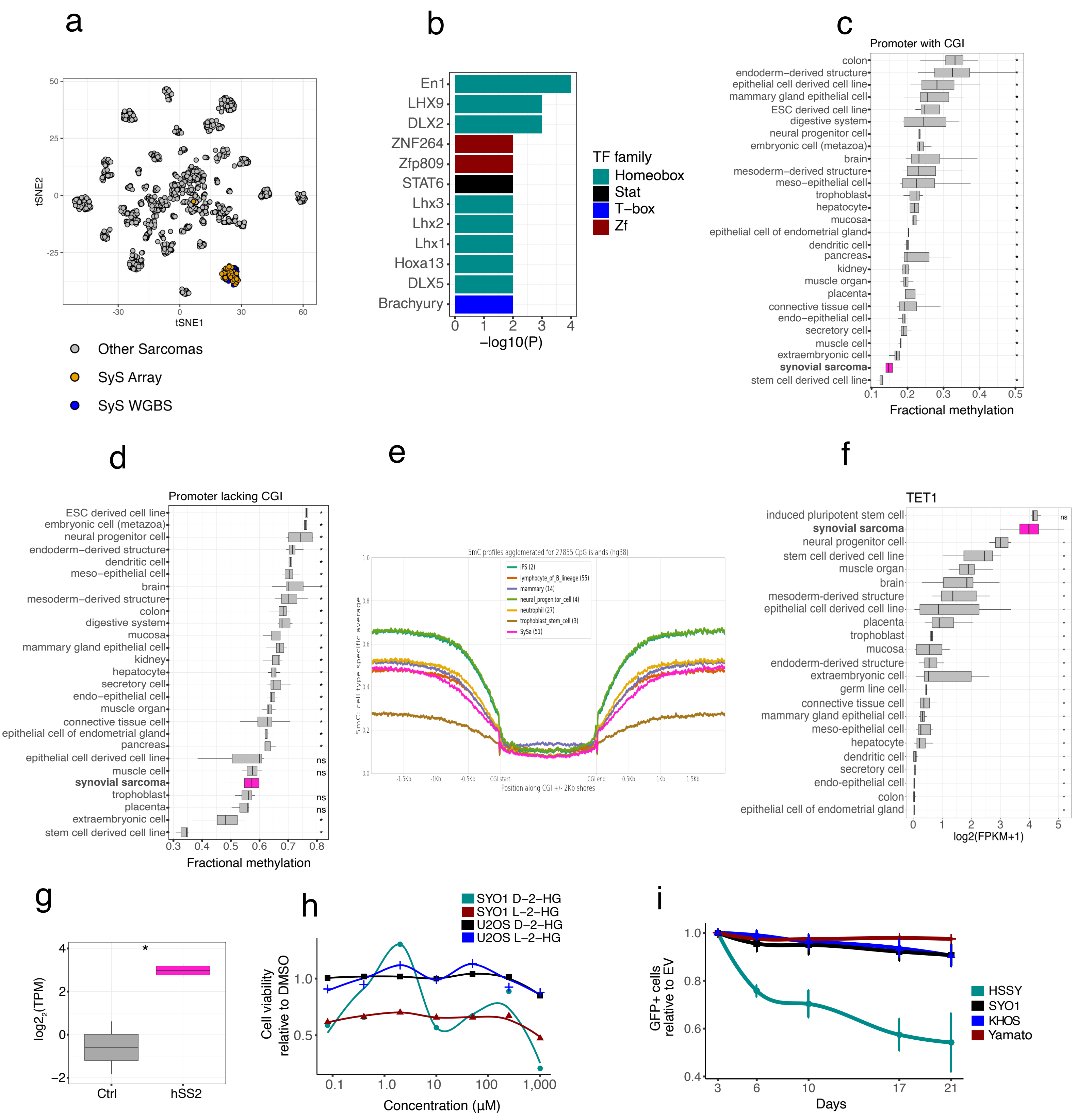

Supplemental Figure 8

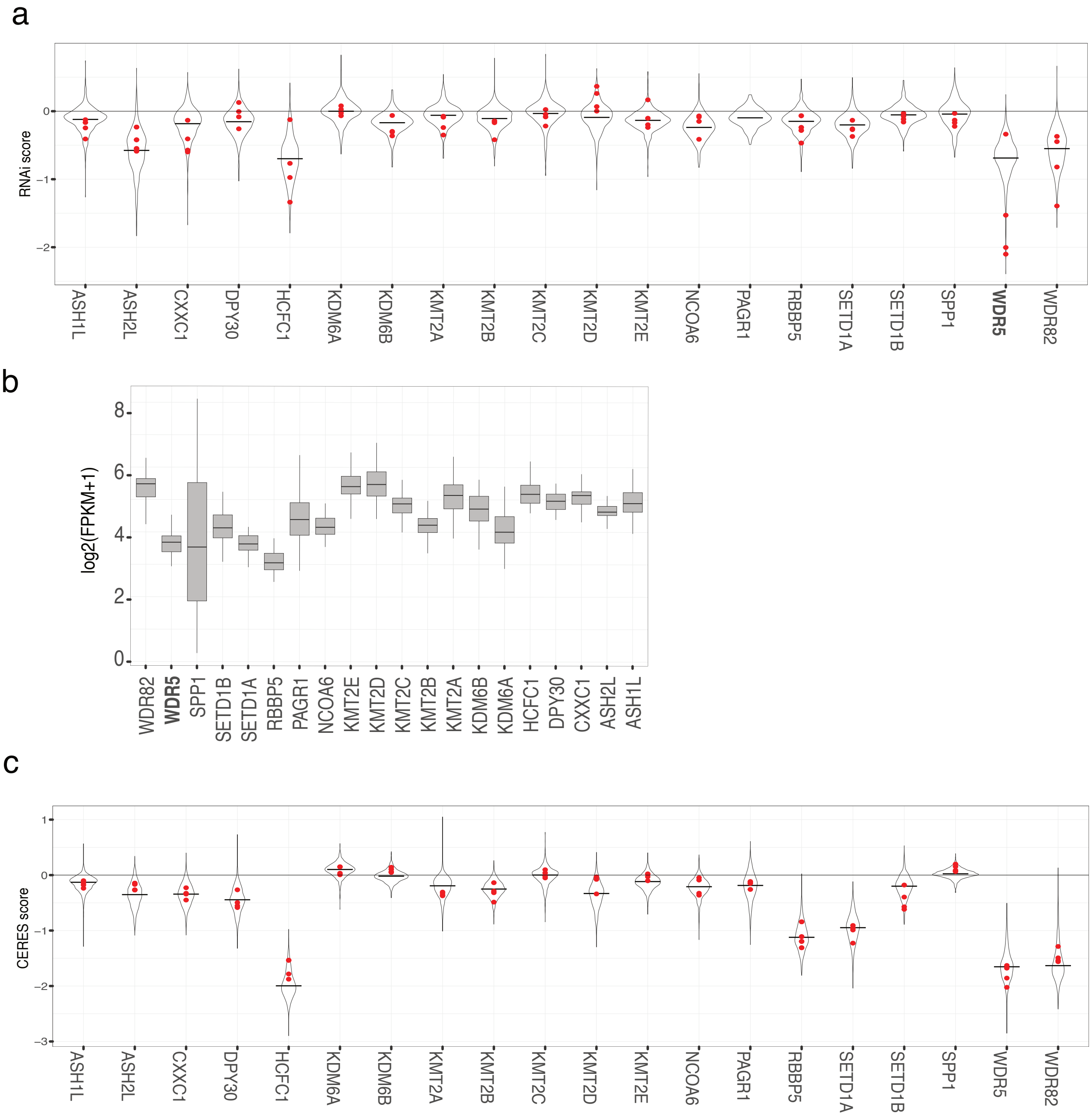

Supplement: Supplement 7 [file NIHPP2024.05.14.594262v1-supplement-7.pdf]
